# Supplementary material for: The splicing factor RBM25 controls MYC activity in acute myeloid leukemia
Source: Nat Commun. 2019 Jan 11;10:172. doi: 10.1038/s41467-018-08076-y (PMC6329799; doi:10.1038/s41467-018-08076-y)
Supplement: Supplementary file 1 — Supplementary Information [file 41467_2018_8076_MOESM1_ESM.pdf]

**Supplementary Information**

**The splicing factor RBM25 controls MYC activity in Acute Myeloid Leukemia**

**Ge et al.**

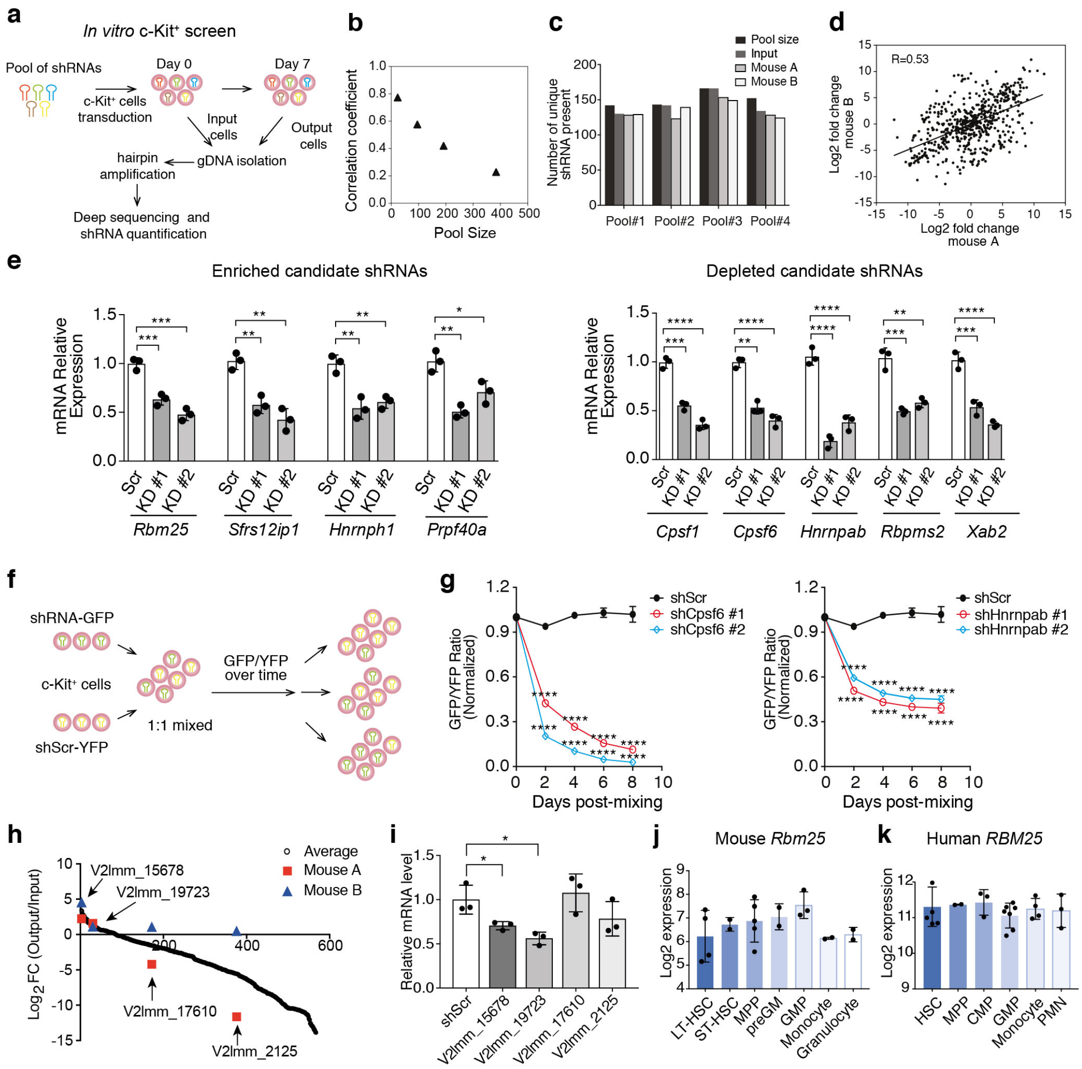


**Supplementary Fig. 1: Related to Fig. 1**

**a** Schematic outline of the *in vitro* screen in c-Kit+ cells. **b** Correlation between the two biological replicates versus different pool sizes. R-values represent Spearman correlation coefficients. **c** Number of unique shRNAs recovered from the *in vivo* Lp30 AML screen after sequencing. **d** Correlation between biological replicates in the *in vivo* Lp30 AML screen in terms of fold-change between input and output samples. R-value represents a Spearman correlation coefficient. **e** Knockdown efficiencies of enriched shRNAs and depleted shRNAs as validated by RT-qPCR. **f** Experimental setup of *in vitro* competitive assay on c-Kit+ cells. **g** Depletion of *Cpsf6* KD cells (left) and *Hnrnpab* KD cells (right) in c-Kit-enriched normal BM cells. h, Performance of individual shRNA targeting *Rbm25* in the *in vivo* Lp30 screen. **i,** *Rbm25* KD efficiency in Lp30 cells transduced with the individual shRNAs. Data represent the mean ± s.d., n = 3. Data were subjected to an unpaired t-test and asterisks indicate the following: *$P$ < 0.05, **$P$ < 0.01, ***$P$ < 0.001, ****$P$ < 0.0001. **j - k** *Rbm25/RBM25* expression in murine and human myeloid differentiation. Data is derived from http://servers.binf.ku.dk/bloodspot/. LT-HSC: Long term Hematopoietic stem cell; ST-HSC: Short term Hematopoietic stem cell; MPP: Multipotential progenitor; preGM: pre-granulocyte monocyte progenitor; GMP: Granulocyte monocyte progenitor; PMN: Polymorphonuclear cells. All replicates in **d**, **g** and **i** are biological replicates.

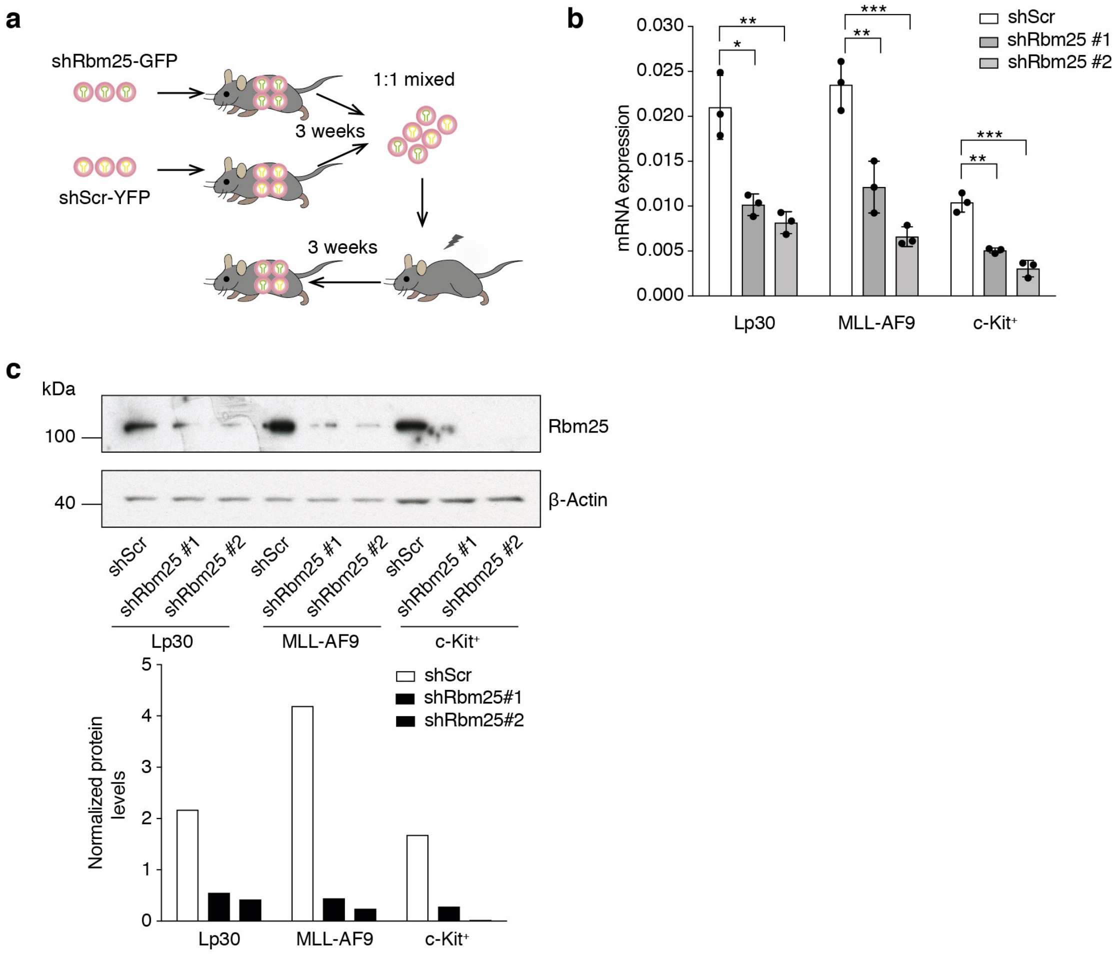


**Supplementary Fig. 2: Related to Fig. 2**

**a** Experimental set-up of the *in vivo* serial competitive bone marrow transplantation assay in **Fig. 2c**. **b** qPCR assay showing the Rbm25 mRNA base line levels and KD efficiency in Lp30 cells, MLL-AF9 cells and cKit+ cells. **c** Western blot showing protein levels (upper panel) in the same cells as shown in **b**. Band intensities were quantified by ImageStudioLite and normalized to β-Actin (lower panel). Data represent the mean ± s.d., n = 3. Data were subjected to an unpaired t-test and asterisks indicate the following: *$P < 0.05$, **$P < 0.01$, ***$P < 0.001$. Panel **b** and **c** show representative results of ≥2 independent experiments. All replicates in **b** and **c** are biological replicates.

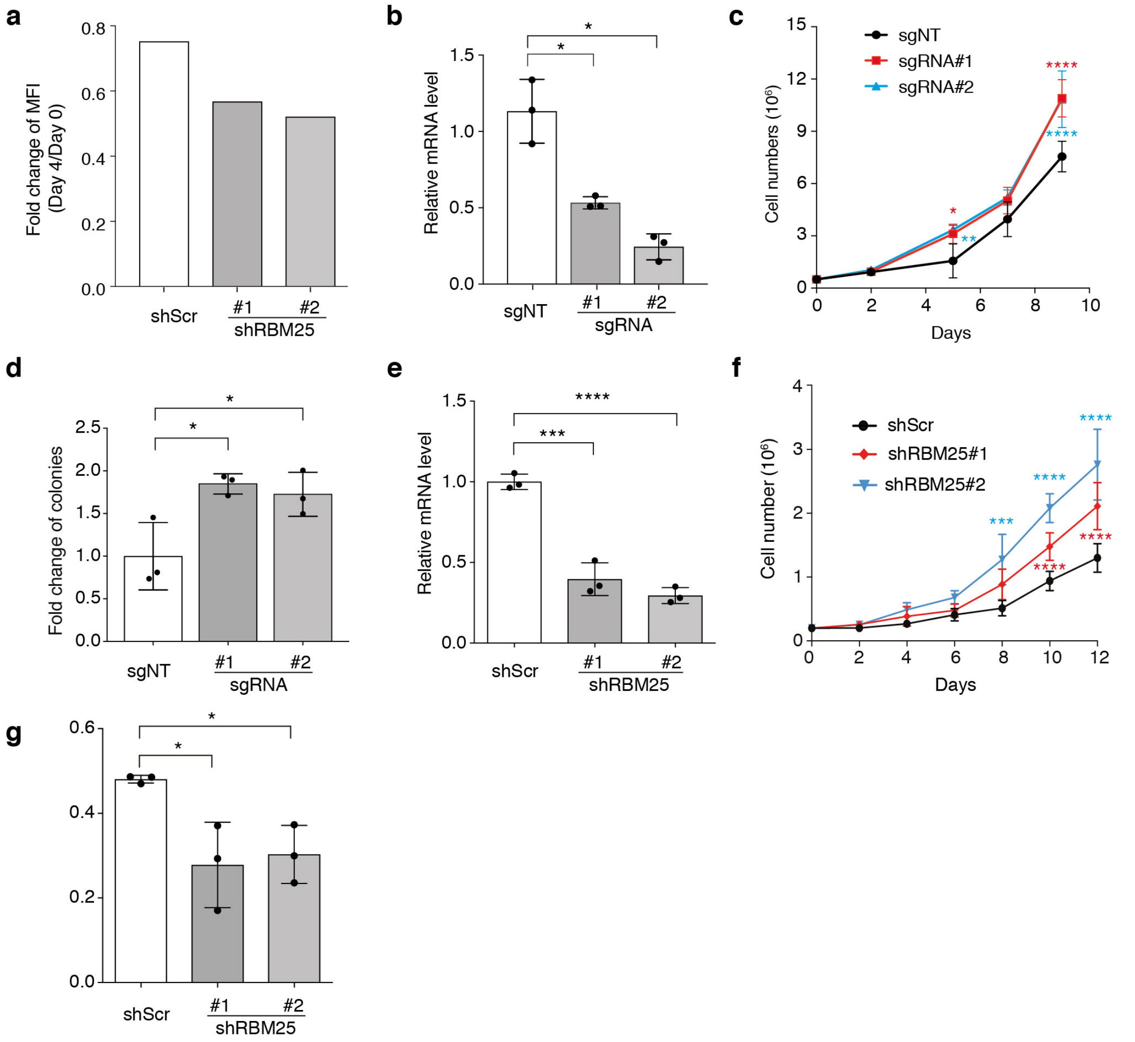


**Supplementary Fig. 3: Related to Fig. 3**

**a** Quantification of the CSFE assay. MFI: Mean florescence intensity. **b** *RBM25* expression levels in U937 cells following CRISPR–(d)Cas9-KRAB-mediated gene silencing. **c** Proliferation of cells from panel **b.** **d** Colony forming capacity of cells from panel **b.** **e** sh-mediated KD of *RBM25* in Kasumi-1 cells. **f** Proliferation of cells from panel **e.** **g** Annexin V/PI staining of cells from panel **e**. Data represent the mean ± s.d., n = 3. Data were subjected to an unpaired t-test and asterisks indicate the following: **P* < 0.05, ***P* < 0.01, ****P* < 0.001, *****P* < 0.0001. All replicates are biological replicates.

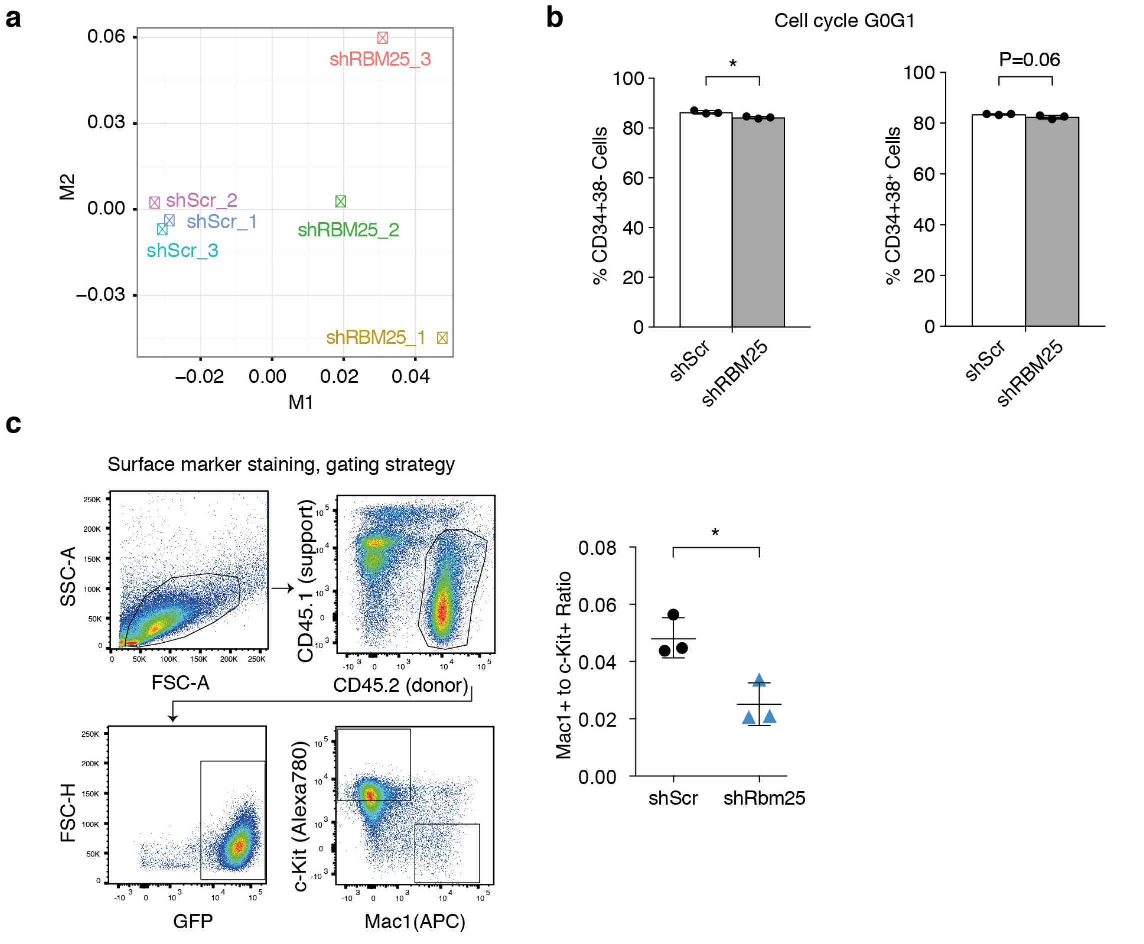


**Supplementary Fig. 4: Related to Fig. 4**

**a** Principal Component Analysis (PCA) plot of control (shScr) and *RBM25* KD (shRBM25) U937 cells based on all detected genes in the RNA-seq data. **b** Proportion of the LSC (left) and progenitor (right) populations of 8227 cells in G0/G1 phase following *RBM25* KD as assessed by PI staining. **c** Effect of *Rbm25* KD on myeloid differentiation of Lp30 cells *in vivo*. Gating strategy (left panel) and quantification (right panel) of c-Kit$^{+}$ -to Mac1$^{+}$ ratios in donor-derived cells of recipients transplanted with cells transduced with either shScr-GFP or shRBM25#2-GFP and FACS sorted for GFP$^{+}$ cells. Data represent the mean ± s.d., n = 3. Data were subjected to an unpaired t-test and asterisks indicate the following: **P* < 0.05. Panel **b** shows representative results of 2 independent experiments. All replicates in **b** and **c** are biological replicates.

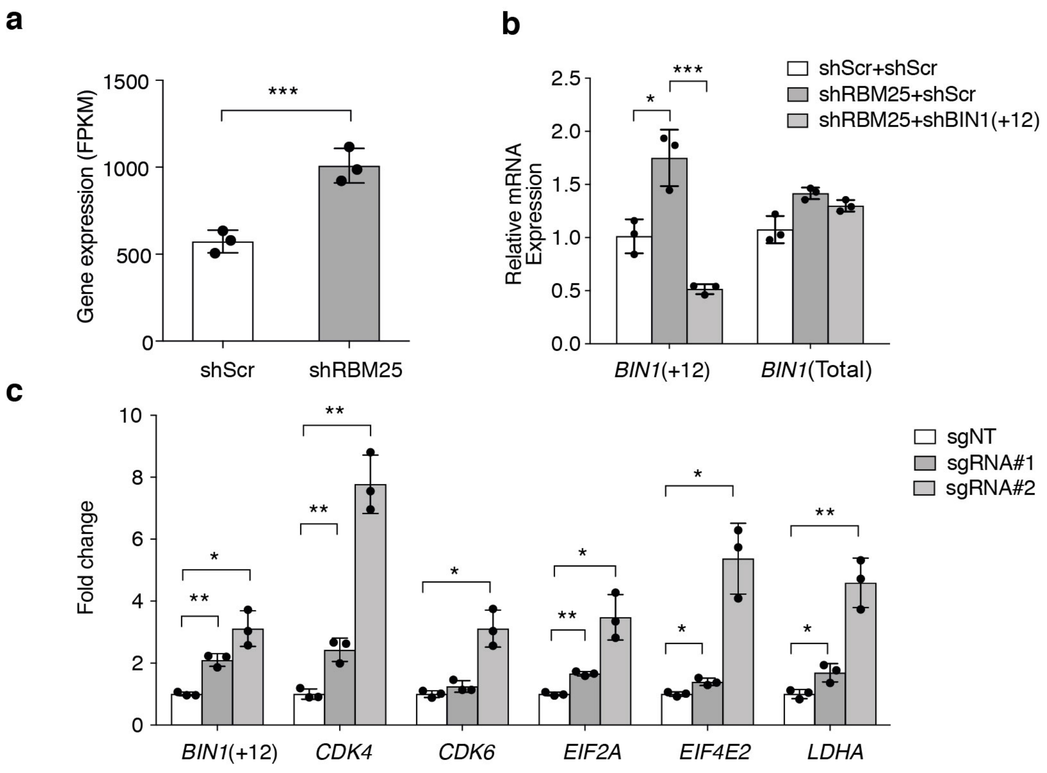


**Supplementary Fig. 5: Related to Fig. 6-7**

**a** Total *BIN1* expression levels in U937 cells before and after *RBM25* KD assessed by RNA-Seq. **b** Expression of *BIN1*(+12) and total *BIN1* transcripts in scrambled, *RBM25* KD and *RBM25/BIN1(+12)* double KD U937 cells. **c** The expression of *RBM25*, *BIN1*(+12) and MYC target genes assayed by qPCR in U937 cells after *RBM25* KD using CRISPRi. Data represent the mean ± s.d., n = 3. Data were subjected to an unpaired t-test and asterisks indicate the following: **P* < 0.05, ***P* < 0.01, ****P* < 0.001. Panel **b** shows representative results of 2 independent experiments. All replicates in **a**, **b** and **c** are biological replicates.

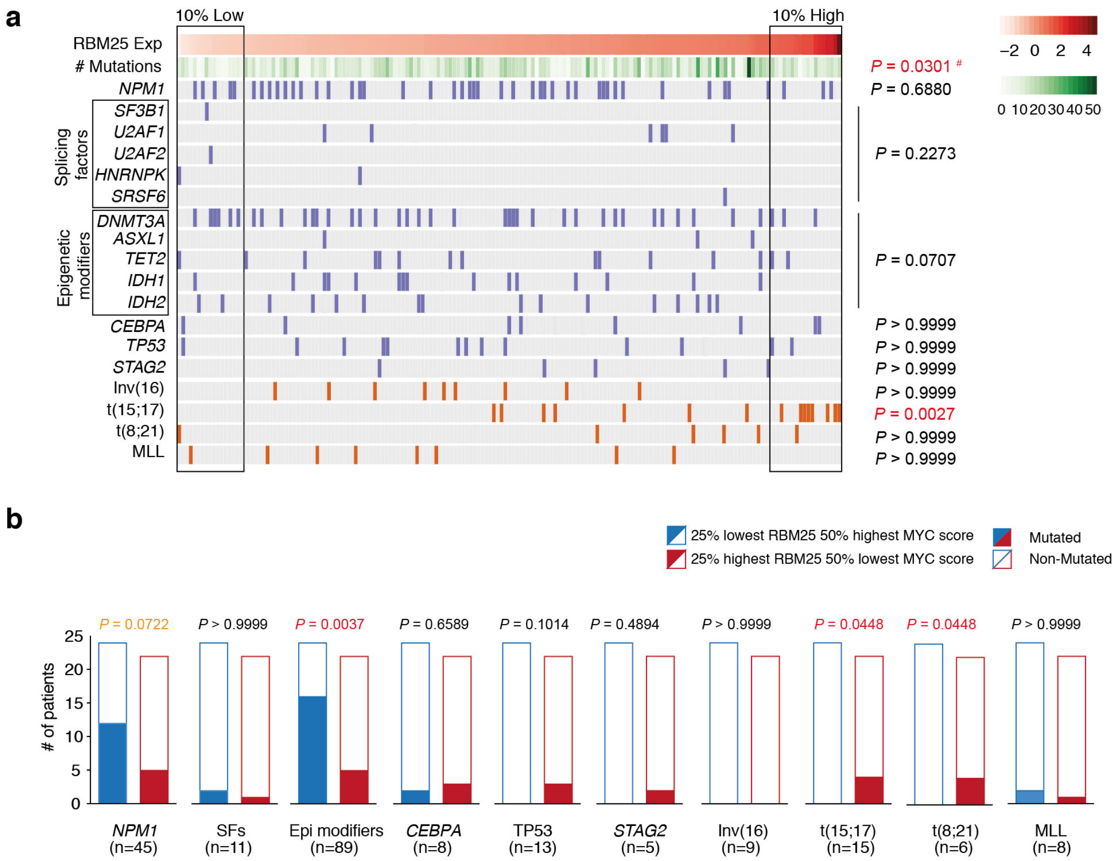


**Supplementary Fig. 6: Related to Fig. 8**

**a** Oncoprint mutation overview of the TCGA AML data set. Frequency of mutations in groups of patients according to *RBM25* expression for the different subtypes were compared by two-sided Fisher exact tests, #calculated using Mann-Whitney test. **b** Mutation profile of patients displaying the 25% lowest *RBM25* levels and highest (above median) MYC target gene score (blue) and patients displaying the 25% highest *RBM25* levels and lowest (beelos median) MYC target gene score (red). The frequencies of mutations in the two patient groups were compared by a two-sided Fisher exact test. n indicates the number of mutations in the entire cohort.

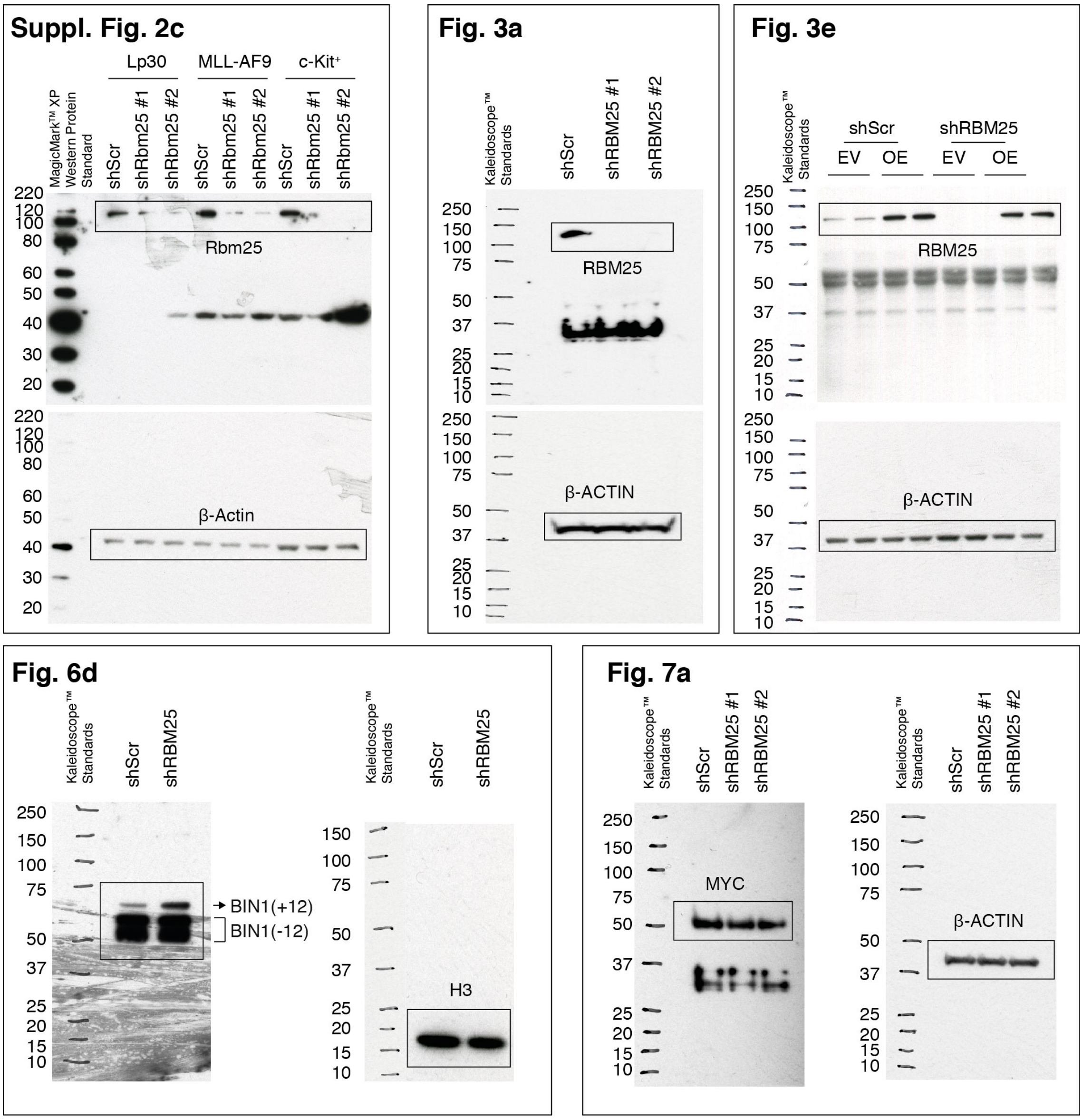


**Supplementary Fig. 7**
Full Western Blots of the blots shown in Suppl. Fig. 2b, Fig. 3a, 3e, 6d and 7a.

**Supplementary Table 1:**

**Enriched candidate shRNAs in the *in vivo* Lp30 screen***

| Library ID | shRNA target gene | *In vivo* Lp30 screen | | *In vitro* c-Kit+ screen | |
|---|---|---|---|---|---|
| | | Rank | Average log2FC (Output/input) | Rank | Average Log2FC (Day7/Day0) |
| v3lmm_496936 | Cdc40 | 31 | 1.36 | 219 | 0.05 |
| v3lmm_496937 | Cdc40 | 112 | -0.54 | 125 | 0.39 |
| v2lmm_176986 | Elavl1 | 13 | 2.24 | 62 | 0.66 |
| v2lmm_200885 | Elavl1 | 47 | 0.70 | 38 | 0.85 |
| v2lmm_74146 | Elavl2 | 28 | 1.41 | 488 | -1.73 |
| v3lmm_433870 | Elavl2 | 40 | 0.95 | 420 | -0.80 |
| v2lmm_65348 | Elavl2 | 46 | 0.73 | 56 | 0.72 |
| v2lmm_104915 | Fubp3 | 36 | 1.15 | 3 | 1.57 |
| v2lhs_77113 | Fubp3 | 101 | -0.30 | 160 | 0.25 |
| v3lmm_503927 | Hnrnph1 | 39 | 0.99 | 17 | 1.10 |
| v2lmm_81803 | Hnrnph1 | 43 | 0.82 | 370 | -0.52 |
| v2lmm_77126 | Hnrnph1 | 98 | -0.21 | 332 | -0.34 |
| v2lmm_191894 | Hnrnpul1 | 50 | 0.63 | 318 | -0.30 |
| v2lmm_30550 | Hnrnpul1 | 70 | 0.32 | 94 | 0.49 |
| v2lmm_177002 | Lsm1 | 77 | 0.26 | 381 | -0.56 |
| v2lmm_176999 | Lsm1 | 100 | -0.27 | 217 | 0.05 |
| v2lmm_78127 | Prpf18 | 69 | 0.33 | 30 | 0.92 |
| v2lmm_66463 | Prpf18 | 104 | -0.37 | 96 | 0.47 |
| v3lmm_452531 | Prpf40a | 20 | 1.65 | 75 | 0.59 |
| v2lmm_4112 | Prpf40a | 26 | 1.50 | 433 | -0.90 |
| v2lmm_1347 | Prpf40a | 34 | 1.30 | 389 | -0.59 |
| v2lhs_174351 | Prpf40a | 71 | 0.32 | 195 | 0.12 |
| v2lmm_10695 | Prpf40a | 89 | -0.13 | 282 | -0.16 |
| v2lmm_80411 | Qk | 72 | 0.31 | 12 | 1.18 |
| v2lmm_150498 | Qk | 15 | 2.16 | 32 | 0.90 |
| v3lmm_417948 | Qk | 88 | -0.10 | 115 | 0.43 |
| v2lmm_150495 | Qk | 106 | -0.40 | 161 | 0.25 |
| v3lmm_461015 | Rbm22 | 61 | 0.53 | 20 | 1.04 |
| v2lmm_202528 | Rbm22 | 76 | 0.27 | 180 | 0.19 |
| v2lmm_15678 | Rbm25 | 3 | 3.40 | 422 | -0.81 |
| v2lmm_19723 | Rbm25 | 30 | 1.37 | 510 | -2.62 |
| v2lmm_7458 | Rbm4 | 17 | 1.99 | 472 | -1.46 |
| v2lmm_11038 | Rbm4 | 45 | 0.77 | 1 | 2.62 |
| v3lmm_442370 | Rod1 | 67 | 0.40 | 124 | 0.39 |
| v3lmm_442373 | Rod1 | 85 | 0.03 | 41 | 0.81 |
| v3lmm_442372 | Rod1 | 102 | -0.34 | 42 | 0.81 |
| v2lmm_67661 | Sfrs12ip1 | 16 | 2.15 | 338 | -0.38 |
| v2lmm_57318 | Sfrs12ip1 | 58 | 0.58 | 377 | -0.55 |
| v2lhs_20638 | Sfrs6 | 44 | 0.79 | 89 | 0.51 |

| | | | | | |
|---|---|---|---|---|---|
| v2lmm_245611 | Sfrs6 | 91 | -0.16 | 67 | 0.63 |
| v2lmm_68400 | Snrnp48 | 38 | 1.07 | 288 | -0.19 |
| v2lmm_79463 | Snrnp48 | 96 | -0.19 | 166 | 0.24 |
| v2lmm_12249 | Syf2 | 60 | 0.53 | 222 | 0.04 |
| v2lmm_8104 | Syf2 | 78 | 0.24 | 164 | 0.25 |
| v2lmm_195598 | Tra2a | 75 | 0.28 | 265 | -0.10 |
| v2lmm_95594 | Tra2a | 90 | -0.13 | 26 | 0.98 |
| v3lmm_434697 | Ttf1 | 6 | 2.89 | 80 | 0.56 |
| v3lmm_434701 | Ttf1 | 12 | 2.25 | 344 | -0.41 |

* Enriched candidates (highlighted in blue) are selected based on the following criteria: 1) multiple shRNAs targeting the candidate gene should be located in the 20 percentile of the most enriched shRNAs (rank < 112 out of 563 shRNAs detected in the screen) from the *in vivo* Lp30 screen and 2) no enrichment of shRNAs targeting the candidate gene should occur *in vitro* c-Kit$^+$ cells screen.

**Supplementary Table 2:**

**Depleted candidate shRNAs in the *in vivo* Lp30 screen***

| Library ID | shRNA target gene | *In vivo* Lp30 screen | | *In vitro* c-Kit+ screen | |
|---|---|---|---|---|---|
| | | Rank | Average log2FC (Output/input) | | Average Log2FC (Day7/Day0) |
| v2lmm_67590 | Cpsf1 | 440 | -7.94 | 206 | 0.09 |
| v2lmm_77783 | Cpsf1 | 503 | -10.43 | 83 | 0.55 |
| v2lmm_194685 | Cpsf6 | 450 | -8.33 | 384 | -0.57 |
| v2lhs_149712 | Cpsf6 | 489 | -9.84 | 346 | -0.41 |
| v2lmm_197085 | Cpsf6 | 488 | -9.83 | 460 | -1.26 |
| v2lmm_99532 | Cpsf6 | 511 | -10.47 | 470 | -1.37 |
| v2lmm_102242 | Ddx23 | 460 | -8.49 | 537 | -6.69 |
| v2lmm_205504 | Ddx23 | 464 | -8.66 | 31 | 0.91 |
| v2lmm_46169 | Fus | 466 | -8.71 | 375 | -0.55 |
| v2lmm_58157 | Fus | 526 | -10.67 | 323 | -0.32 |
| v2lmm_131652 | Hnrnpa0 | 442 | -8.02 | 386 | -0.58 |
| v2lmm_131650 | Hnrnpa0 | 474 | -9.06 | 175 | 0.20 |
| v2lmm_176047 | Hnrnpa2b1 | 429 | -7.65 | 491 | -1.81 |
| v2lmm_194016 | Hnrnpa2b1 | 510 | -10.47 | 84 | 0.54 |
| v2lmm_71197 | Hnrnpab | 439 | -7.92 | 186 | 0.15 |
| v2lmm_82139 | Hnrnpab | 523 | -10.65 | 359 | -0.48 |
| v2lhs_149883 | Sfrs1 | 447 | -8.10 | 468 | -1.36 |
| v2lhs_202414 | Sfrs1 | 449 | -8.22 | 540 | -7.15 |
| v3lmm_484329 | Sfrs1 | 562 | -13.44 | 520 | -3.38 |
| v2lhs_149884 | Sfrs1 | 563 | -13.47 | 519 | -3.18 |
| v2lmm_23430 | Lsm4 | 473 | -9.01 | 539 | -6.86 |
| v3lmm_431893 | Lsm4 | 487 | -9.78 | 232 | 0.01 |
| v2lmm_150317 | Nova1 | 525 | -10.66 | 157 | 0.25 |
| v2lmm_150318 | Nova1 | 531 | -10.81 | 390 | -0.61 |
| v2lmm_191818 | Nova1 | 533 | -10.84 | 499 | -2.16 |
| v2lmm_51989 | Prpf38a | 517 | -10.55 | 533 | -4.97 |
| v2lmm_59672 | Prpf38a | 542 | -11.16 | 401 | -0.67 |
| v3lmm_425253 | Puf60 | 482 | -9.43 | 530 | -4.55 |
| v3lmm_425254 | Puf60 | 567 | -13.87 | 538 | -6.77 |
| v2lmm_18299 | Rbpms2 | 513 | -10.50 | 302 | -0.25 |
| v2lmm_4825 | Rbpms2 | 512 | -10.48 | 101 | 0.46 |
| v2lmm_64078 | Sf3a1 | 457 | -8.44 | 525 | -3.87 |
| v2lmm_79143 | Sf3a1 | 559 | -12.97 | 498 | -2.15 |
| v3lmm_463990 | Sf3b2 | 435 | -7.74 | 436 | -0.93 |
| v2lmm_85024 | Sf3b2 | 479 | -9.31 | 178 | 0.20 |
| v2lmm_85026 | Sf3b2 | 561 | -13.17 | 512 | -2.74 |
| v3lmm_463993 | Sf3b2 | 565 | -13.67 | 503 | -2.27 |
| v2lmm_54487 | Sfrs10 | 530 | -10.80 | 287 | -0.18 |
| v2lhs_263160 | Sfrs10 | 468 | -8.76 | 466 | -1.34 |

| | | | | | |
|---|---|---|---|---|---|
| v2lmm_36106 | Sfrs10 | 557 | -12.91 | 516 | -2.85 |
| v2lmm_64640 | Sltm | 521 | -10.63 | 19 | 1.05 |
| v2lmm_73435 | Sltm | 527 | -10.69 | 486 | -1.69 |
| v2lmm_84277 | Taf15 | 546 | -11.34 | 329 | -0.33 |
| v2lhs_172494 | Taf15 | 556 | -12.86 | 404 | -0.70 |
| v2lmm_61929 | Xab2 | 421 | -7.30 | 133 | 0.36 |
| v2lmm_220955 | Xab2 | 423 | -7.36 | 283 | -0.17 |
| v2lmm_72510 | Xab2 | 494 | -10.14 | 531 | -4.61 |

* Depleted candidates (highlighted in blue) are selected based on the following criteria: 1) multiple shRNAs targeting the candidate gene should be located in the 25 percentile of the most depleted shRNAs (rank >421 out of 563 shRNAs detected in the screen) from the *in vivo* Lp30 screen and 2) no depletion of shRNAs targeting the candidate gene should occur *in vitro* c-Kit$^{+}$ cells screen.

**Supplementary Table 3: Primers carrying Illumina adaptors and barcodes**

| | |
|---|---|
| Illumina_notag_F_PE2.0 § | CAAGCAGAAGACGGCATACGAGATCGGTCTCGGCATTCCTGCTGAACCGCTCTTCCGATCTNNNNNGGTAATTGTTTGAATGAGGC |
| llumina_GT_R_PE1.0 | AATGATACGGCGACCACCGAGATCTACACTCTTTCCCTACACGACGCTCTTCCGATCTNNNNNGTCCTTGAATTCCGAGGCAGTAGGCA |
| llumina_AA_R_PE1.0 | AATGATACGGCGACCACCGAGATCTACACTCTTTCCCTACACGACGCTCTTCCGATCTNNNNNAACCTTGAATTCCGAGGCAGTAGGCA |
| llumina_TT_R_PE1.0 | AATGATACGGCGACCACCGAGATCTACACTCTTTCCCTACACGACGCTCTTCCGATCTNNNNNTTCCTTGAATTCCGAGGCAGTAGGCA |
| llumina_CC_R_PE1.0 | AATGATACGGCGACCACCGAGATCTACACTCTTTCCCTACACGACGCTCTTCCGATCTNNNNNCCCCTTGAATTCCGAGGCAGTAGGCA |
| llumina_GG_R_PE1.0 | AATGATACGGCGACCACCGAGATCTACACTCTTTCCCTACACGACGCTCTTCCGATCTNNNNNGGCCTTGAATTCCGAGGCAGTAGGCA |
| llumina_AT_R_PE1.0 | AATGATACGGCGACCACCGAGATCTACACTCTTTCCCTACACGACGCTCTTCCGATCTNNNNNATCCTTGAATTCCGAGGCAGTAGGCA |
| llumina_TA_R_PE1.0 | AATGATACGGCGACCACCGAGATCTACACTCTTTCCCTACACGACGCTCTTCCGATCTNNNNNTACCTTGAATTCCGAGGCAGTAGGCA |
| llumina_TC_R_PE1.0 | AATGATACGGCGACCACCGAGATCTACACTCTTTCCCTACACGACGCTCTTCCGATCTNNNNNTCCCTTGAATTCCGAGGCAGTAGGCA |
| llumina_AG_R_PE1.0 | AATGATACGGCGACCACCGAGATCTACACTCTTTCCCTACACGACGCTCTTCCGATCTNNNNNAGCCTTGAATTCCGAGGCAGTAGGCA |
| llumina_GA_R_PE1.0 | AATGATACGGCGACCACCGAGATCTACACTCTTTCCCTACACGACGCTCTTCCGATCTNNNNNGACCTTGAATTCCGAGGCAGTAGGCA |
| llumina_AC_R_PE1.0 | AATGATACGGCGACCACCGAGATCTACACTCTTTCCCTACACGACGCTCTTCCGATCTNNNNNACCCTTGAATTCCGAGGCAGTAGGCA |
| llumina_CA_R_PE1.0 | AATGATACGGCGACCACCGAGATCTACACTCTTTCCCTACACGACGCTCTTCCGATCTNNNNNCACCTTGAATTCCGAGGCAGTAGGCA |

§ Illumina_notag_F_PE2.0 was used in combination with either of the Illumina_R_PE1.0

**Supplementary Table 4: Oligonucleotides used for cloning**

| Gene name | Oligo Sequences (5'-3') |
|---|---|
| RBM25 gRNA#1 Forward | CACCGAACTGCGGGGGTGGGATCCC |
| RBM25 gRNA#1 Reverse | AAACGGGATCCCACCCCCGCAGTTC |
| RBM25 gRNA#2 Forward | CACCGTCATTTGAATCGCCCTCCCA |
| RBM25 gRNA#2 Reverse | AAACTGGGAGGGCGATTCAAATGAC |
| shBIN1(+12) Forward | AATTAGCAGATCCTCAGCCTGTTTGCTCGAGCAAACAGGCTGAGGATCTGCTTTTTTTTAT |
| shBIN1(+12) Reverse | AAAAAAAAGCAGATCCTCAGCCTGTTTGCTCGAGCAAACAGGCTGAGGATCTGCT |
| shMYC Forward | AATTCCTGAGACAGATCAGCAACAACTCGAGTTGTTGCTGATCTGTCTCAGGTTTTTTTAT |
| shMYC Reverse | AAAAAAACCTGAGACAGATCAGCAACAACTCGAGTTGTTGCTGATCTGTCTCAGG |
| RBM25_R Forward | CCGGGTTTTCTTTCGTTCTCTGATCTCCCAGTTTTTGAGACGCTCTTGATAAGCAGCTTCTTTC |
| RBM25_R Reverse | GAAAGAAGCTGCTTATCAAGAGCGTCTCAAAAACTGGGAGATCAGAGAACGAAAGAAAACCCGG |

**Supplementary Table 5: qPCR primer Sequences**

| Target gene | Forward primer (5'-3') | Reverse primer (5'-3') |
|---|---|---|
| *Rbm25* | TCACCACAGAGCATTTTAGATGA | CTGTTTCATATATCAATAATCTCCACA |
| *Hnrnph1* | AGCGGTGGTGCTTATGGTAG | GCCACCGTAACTGGACTGG |
| *Sfrs12ip* | AAGGAAAAGAGCAGGGAGAAA | TTTGCGGAGTCCTCTTCAGT |
| *Prpf40a* | CCTAAGAAAAAGACTGGAAAGGA | TCCAATTCCCCTTCACTCAG |
| *Cpsf6* | CTCTCTGCAGGATTGCCTTC | GTCCCGTTCTCTTGATCGTT |
| *Hnrnpab* | CAGAGGAAATCGCAATCGAG | GCTGGCTCTTTCCGTAATTT |
| *Cpsf1* | CAGTGAGCTGGCCAAGAAG | TGTCTCCAGCAAGTCATCCA |
| *Rbpms2* | CACCTACCCAGCTGCCACT | GTGGTGTCAGAGGAGGGGTA |
| *Xab2* | ATGAGGACGAGGACGAGATG | CTTCCTTTAGGCTCCCGAAC |
| *Actg* | CTCTTCCAGCCTTCCTTCCT | TGCTAGGGCTGTGATCTCCT |
| *RBM25* | TGTCTTTTCCACCTCATTTGAATCG | ATTGGTACAGGAATCATTGGGGT |
| *BCL2L1-L* | TAAACTGGGGTCGCATTGTG | AGGTAAGTGGCCATCCAAGC |
| *BCL2L1-S* | GCAGTAAAGCAAGCGCTGAG | GTTCCACAAAAGTATCCTGTTCAAAG |
| *BIN1*(Ex11-14)# | CCTCCAGATGGCTCCCCTGC | CCCGGGGGCAGGTCCAAGCG |
| *BIN1*(EX12) | AAACACACCCCGTCCAAG | ACGCTGATCTCAGGGACAAA |
| *BIN1*(EX4-5)$ | GGCTTCCAAGAAGCTGAATG | GGTGGTAATCCATCCACAGC |
| *CDK4* | GGCCCTCAAGAGTGTGAGAG | CTGGTCGGCTTCAGAGTTTC |
| *CDK6* | CCGTGGATCTCTGGAGTGTT | TCTCCTGGGAGTCCAATCAC |
| *EIF2A* | AGGTGAAGAGGTTGGGGACT | TACAGCTGGCTGCTTTCTCA |
| *EIF4E2* | ATGATGACAGTGGGGACCAT | GTTGTACTGCAGGGGATGCT |
| *LDHA* | TGTGCCTGTATGGAGTGGAA | AGCACTCTCAACCACCTGCT |
| *CAD* | ACGGAGCTGACGGAAGATTA | CTCGTGAAATGCACTGAGGA |
| *MYC* | GCTGTTTGAAGGCTGGATTTC | GATGAAATAGGGCTGTACGGAG |
| *GAPDH* | AATCCCATCACCATCTTCCA | TGGACTCCACGACGTACTCA |

# Primer pairs used for RT-PCR (Fig. 6c)

$ Primer pairs used for assessment of total BIN1 expression (Supplementary Fig. 5b)
