# Supplementary material for: The splicing factor RBM25 controls MYC activity in acute myeloid leukemia
Source: Nat Commun. 2019 Jan 11;10:172. doi: 10.1038/s41467-018-08076-y (PMC6329799; doi:10.1038/s41467-018-08076-y)
Supplement: Supplementary file 3 — Description of Additional Supplementary Files [file 41467_2018_8076_MOESM3_ESM.docx]

**Title:** Supplementary Data 1:
**Description:** Splicing factor library; Raw read counts (Lp30 screen); Raw read counts (cKit screen)

**Title:** Supplementary Data 2:
**Description:** Differentially expressed genes between shRBM25 and shScr transduced U937 cells

**Title:** Supplementary Data 3:
**Description:** Differentially expressed isoforms between shRBM25 and shScr transduced U937 cells
